# Digital Tools to Support Family-Based Weight Management for Children: Mixed Methods Pilot and Feasibility Study

**DOI:** 10.2196/24714

**Published:** 2021-01-07

**Authors:** Amanda E Staiano, Jenelle R Shanley, Holly Kihm, Keely R Hawkins, Shannon Self-Brown, Christoph Höchsmann, Melissa C Osborne, Monique M LeBlanc, John W Apolzan, Corby K Martin

**Affiliations:** 1 Pennington Biomedical Research Center Baton Rouge, LA United States; 2 Pacific University Hillsboro, OR United States; 3 Southeastern Louisiana University Hammond, LA United States; 4 IDEA Public Schools Austin, TX United States; 5 Georgia State University Atlanta, GA United States

**Keywords:** parent training, weight loss, telehealth, obesity, SafeCare

## Abstract

**Background:**

Family-based behavioral therapy is an efficacious approach to deliver weight management counseling to children and their parents. However, most families do not have access to in-person, evidence-based treatment. We previously developed and tested DRIVE (Developing Relationships that Include Values of Eating and Exercise), a home-based parent training program to maintain body weight among children at risk for obesity, with the intent to eventually disseminate it nationally alongside SafeCare, a parent support program that focuses on parent-child interactions. Currently the DRIVE program has only been tested independently of SafeCare. This study created the “mHealth DRIVE” program by further adapting DRIVE to incorporate digital and mobile health tools, including remotely delivered sessions, a wireless scale that enabled a child-tailored weight graph, and a pedometer. Telehealth delivery via mHealth platforms and other digital tools can improve program cost-effectiveness, deliver long-term care, and directly support both families and care providers.

**Objective:**

The objective of this study was to examine preliminary acceptability and effectiveness of the mHealth DRIVE program among children and parents who received it and among SafeCare providers who potentially could deliver it.

**Methods:**

Study 1 was a 13-week pilot study of a remotely delivered mHealth family-based weight management program. Satisfaction surveys were administered, and height and weight were measured pre- and post-study. Study 2 was a feasibility/acceptability survey administered to SafeCare providers.

**Results:**

Parental and child satisfaction (mean of 4.9/6.0 and 3.8/5.0, respectively) were high, and children’s (N=10) BMI z-scores significantly decreased (mean –0.14, SD 0.17; *P*=.025). Over 90% of SafeCare providers (N=74) indicated that SafeCare families would benefit from learning how to eat healthily and be more active, and 80% of providers reported that they and the families would benefit from digital tools to support child weight management.

**Conclusions:**

Pediatric mHealth weight management interventions show promise for effectiveness and acceptability by families and providers.

**Trial Registration:**

Clinicaltrials.gov NCT03297541, https://clinicaltrials.gov/ct2/show/NCT03297541.

## Introduction

Obesity affects nearly one in five children and adolescents in the US [1]. The US Preventive Services Task Force [2] and the American Medical Association [3] recommend comprehensive, intensive, family-based weight management programs to treat childhood obesity. Family-based behavioral therapy is efficacious [4], although most children do not have access to evidence-based treatment due to limited availability of programs and trained providers, barriers for travelling to in-person sessions including transportation and time constraints, and cost of participation due to limited or no insurance coverage [5,6].

To overcome barriers to access, evidence-based models that include parent training (eg, SafeCare, Parents as Teachers) can be delivered in the family’s home [7]. SafeCare is a parent support program delivered by trained providers that focuses on parent-child interactions to mitigate the risk of abuse or neglect. SafeCare is predominantly delivered in the home, but sessions can also be delivered via technology, over video chat and telephone [8]. SafeCare has been disseminated in more than 25 US states and internationally. Currently, there are approximately 100 SafeCare accredited agencies where providers serve more than 6000 families per year. The underlying principles of SafeCare on improving parent-child interaction, coupled with its broad reach to at-risk and underserved families, make SafeCare an ideal platform for delivery of weight management services.

We developed a parent support focused program to treat childhood obesity that can be delivered in the home called DRIVE (Developing Relationships that Include Values of Eating and Exercise) [9] with the intent to eventually disseminate the program across the SafeCare network. DRIVE incorporates SafeCare principles to promote healthy eating, physical activity, and healthy weight in children by fostering positive parent-child interactions. Previously, we tested the efficacy of DRIVE in a 19-week randomized controlled pilot trial in 16 parent/child dyads (children ages 2-6 years with BMIs ≥ 75th percentile) and found that the change in children’s BMI z-scores (BMIz) (Mean –0.1, SE 0.1) was significantly different (*P*<.01) compared to a health education control group (mean 0.5, SE 0.1) [9].

Although DRIVE was initially developed for in-person delivery, telehealth delivery via mHealth platforms can improve cost-effectiveness, deliver long-term care, and directly support both families and care providers [10]. Identifying alternate avenues for families to access care is increasingly important [10], including for children with obesity during the COVID-19 pandemic when families are unwilling or unable to present in-person for treatment [11]. To this end, the objectives of the studies reported herein were to examine 1) the acceptability of a remotely delivered weight management program (mHealth DRIVE) as determined by the parents and children who used the program, 2) the preliminary effectiveness of this virtual program to reduce child body mass, and 3) the perceived need and willingness to deliver mHealth DRIVE by SafeCare providers.

## Methods

### Study 1

#### Participants

Parents were recruited from their children’s after-school wellness program. Parents were invited to attend an informational session that explained the purpose of mHealth DRIVE. Eligibility criteria for children included ages 5 to 14 years; be physically capable of exercise; and be free of diseases that affect metabolism, body weight, and food intake, including type 1 or type 2 diabetes, HIV/AIDS, and cancer. Children were excluded if they had significant cardiovascular disease or disorders or other significant medical problems that would prevent them from engaging in regular physical activity. Inclusion criteria for parents included having a smart phone and being willing to use the smartphone for the intervention. Eleven child/parent dyads enrolled, but 1 dyad was excluded from all analyses because of the child’s low BMI percentile (4^th^ percentile). Parents provided written informed consent, and children provided assent. Study procedures were approved by the Pennington Biomedical Research Center institutional review board.

#### Intervention Sessions, Treatment Goals, and Tracking of Weight and Behaviors

Child/parent dyads attended 8 counseling sessions (approximately 30 minutes each) primarily over their internet-connected device (eg, smartphone, tablet, laptop, or desktop computer). Most interactions were via video calls or phone, but email and text communication also occurred. The DRIVE curriculum was shortened to 13-weeks to align with the school semester. A Pennington Biomedical counselor delivered sessions and provided individualized advice and problem-solving strategies for the parent and the child. Each session included an interactive component for the parent and child related to healthy eating and active play, and interactive parenting training. Sessions were based on treatment methods that promote child weight loss that have been sustained for 10 years [12,13]. Although the sessions were remotely delivered, counselors were able to deploy motivational interviewing techniques to address decreases in motivation, which are inevitable in longer-term interventions [14].

The guiding principles of the sessions were 1) weight and activity monitoring; 2) building commitment and overcoming barriers to healthy behavior changes, with a goal of teaching the parent to model appropriate diet and physical activity behaviors for their child; 3) review of progress and problem-solving to address poor adherence to behavioral goals; and 4) food monitoring and goal setting for nutrient intake. Sessions focused on how to motivate the child and manage noncompliance; techniques included praise and reward, positive reinforcement, selective ignoring, contracting, preplanning for meals and physical activity, shaping behaviors, modeling, changes to the home environment, and facilitating social support for behavior change [15,16]. The dietary approach employed food monitoring and goal setting for nutrient intake, and the Traffic Light Diet [12] was included to facilitate remote modification of dietary changes. The Traffic Light Diet teaches parents and children to categorize foods based on green (low calorie foods to be eaten freely), yellow (moderate-calorie foods to be eaten occasionally), and red (high-calorie foods to be eaten rarely), with the goal to gradually reduce the number of red foods eaten each week. The physical activity approach introduced free or inexpensive activity options that the children enjoyed and addressed barriers to physical activity.

Children’s energy requirements were estimated using a physical activity level of 1.4 and the Harris-Benedict equation, which has good accuracy in youth with obesity [17]. The energy intake goal was 250 kcal/d less than estimated energy requirements, which should promote modest weight loss and weight gain attenuation over time. The activity goal of children was to gradually increase physical activity to a goal of approximately 6,000 steps/day above their personal baseline values, which is appropriate as we expected low baseline physical activity [18]. This activity goal was the equivalent of an additional 30 min/day of moderate-to-vigorous physical activity as a gradual increase towards the physical activity guidelines of 60 min/day of moderate-to-vigorous physical activity [18].

**Figure 1:**
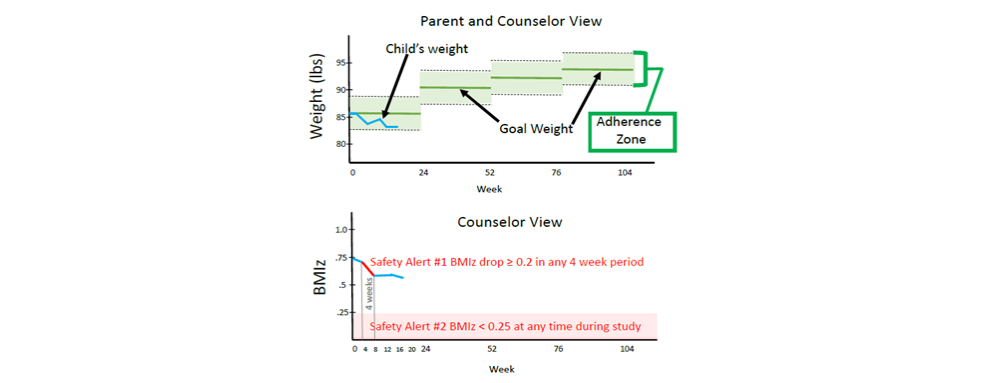
Weight graph zone of child’s adherence. BMIz: BMI z-score.

The intervention content and parent-training approach were based on DRIVE, and the mHealth aspects of the intervention were based on a successful weight management intervention for adults called SmartLoss [19,20]. Specifically, children’s daily physical activity (steps/day) was tracked with a hip-worn Omron HJ-324U pedometer (Omron Healthcare, Inc, Kyoto, Japan), and the parent was asked to document their child’s steps daily. The counselor plotted the child’s daily step data in relation to their individual goals to help promote adherence to activity goals. The children also received a BodyTrace scale that automatically sent their weights to a website accessible by the counselors. Children were asked to weigh themselves at least weekly, unless contraindicated due to anxiety or other mental health barriers, similar to SmartLoss [19,20]. Weighing at the same time of day and in the same state was encouraged, preferably after getting out of bed in the morning and after voiding. Children’s body weight and a weight graph were used to guide intervention delivery to facilitate healthy weight management and avoid unsafe changes in body weight. Specifically, and as detailed in the upper panel of Figure 1, a 6-pound “zone” of acceptable weights or “adherence” was created, and children’s individual weights were plotted against this zone. Hence, the zone promotes weight maintenance, but it allows for weight loss of less than 3 pounds if the child’s BMI is greater than or equal to the 85th percentile. Further, this approach includes objective safety criteria that are triggered if rapid or excessive weight loss occurs (see Figure 1, lower panel).

The program encourages healthy eating, activity, and weight tracking over time. The counselor and parent utilized the weight graph to modulate intervention intensity and as an objective indicator of the need to change the child’s energy intake level. Specifically, the counselor and parent 1) *increase* energy intake if weight loss is excessive, defined as more than 0.2 BMIz reduction within 1 month, which aligns with American Medical Association recommendations for maximum 2 lb/week weight loss in children [3]; 2) *maintain* energy intake if weight maintenance is observed, until the child’s BMIz reaches 0.25 (approximately equivalent to the 60th BMI percentile); and 3) *reduce* energy intake if the child is gaining weight at a rate that increases the child’s BMIz, unless he/she has reached 0.25 BMIz or approximately the 60th percentile, at which time the child increases body weight over time to maintain 0.25 BMIz or approximately the 60th percentile. The threshold of 0.25 BMIz to begin weight maintenance aligns with the goal of reducing BMIz without promoting energy restriction that could negatively impact growth and development. In a longer-term intervention, the zone would be adjusted every 6 months according to increases in the child’s height (see Figure 1), but this pilot study did not adjust the zone due to the study being only 13 weeks in duration. The counselor electronically provided the parent with the child’s weight graph during each session (see the upper panel of Figure 1).

Parents who needed help modifying their child’s diet had the option of sending their counselor images of how they prepare foods and what foods they provide to their child and family. These were not outcome data but provided the counselor with near real-time data on changes the parents could make to improve their child’s diet and health. These images can be captured with any camera-enabled device, and smartphone apps are available to streamline this process (eg, SmartIntake).

#### Measures and Data Analysis

Parents and children completed an acceptability survey at the end of the intervention that included Likert scales on intervention satisfaction (see Table 1). Children’s height and weight (shoes removed, no outer clothing) were collected in duplicate at baseline and end of study by trained assessors, and both assessments occurred in the afternoon. Height was measured with a stadiometer, with the child standing feet flat, with heels, buttocks, upper back, and back of head contacting the stadiometer, and the child’s head facing straight ahead. Weight was measured with a digital scale with the child standing in the middle of the scale with arms hanging loosely at their side. Height and weight were recorded to the nearest 0.1 unit (cm or kg, respectively); if the two measures differed by more than 0.5 units, a third measurement was taken and the closest two of three were used in analysis. Mean values and percentages were calculated for satisfaction surveys. Differences in BMI were examined using *t*-tests, with an alpha level of .05. Analyses were conducted using SPSS.

### Study 2

A survey of SafeCare providers was conducted across the US to assess 1) the perceived need for diet, physical activity, and weight management services for SafeCare children, and 2) the willingness of SafeCare providers to offer such services.

#### Participants

Eighty-two SafeCare providers from 14 states provided consent and completed the survey. The sample was predominantly female (n=71), with 5 males, 1 other, and 5 unknown. The mean age of providers was 39.8 years (SD 12.9), with 17 unknown age data. Thirty-eight providers reported delivering care in urban cluster/suburban areas (2500-50,000 people), 22 in urban areas (≥50,000 people), and 18 in rural areas (<2,500 people), with 4 unknown.

#### Procedures

A recruitment email was sent to the potential participants using the list of contact information for US SafeCare providers. The email contained an anonymous link to the survey conducted through Qualtrics, a secure web-based survey platform that employs high-level security measures to ensure data are protected from malicious data breaches and requires a password in order to download the data. A reminder email was sent 1 week later, reminding participants of the opportunity to complete the survey. The survey was open for 2 weeks.

#### Measures and Data Analysis

The survey queried demographic data (age, gender, and level of urbanicity where services are delivered) and assessed if SafeCare providers perceive a need for or have experience with additional educational material for child nutrition/weight management. Data were cross-sectional and were analyzed descriptively (ie, percentages were reported for categorical variables; means or percentages were reported for Likert scale items). Reported percentages collapse the “Strongly Agree” and “Agree” responses.

## Results

### Study 1

Of the 10 children, 6 were girls (60%) and the mean age was 7.8 years (SD 2.3 years; range 6-14 years). Mean BMI percentile and BMIz were 86th (SD 0.17) and 1.4 (SD 0.7), respectively. Four children had obesity, 4 were overweight, and 2 were normal weight. There was a statistically significant reduction in children’s BMIz over the 13-week period (mean –0.14, SD 0.17; *P*=.025). There was also a significant BMIz reduction among the 8 children who were overweight or had obesity (mean –0.18, SD 0.15; *P*=.013). The 2 normal weight children did not lose weight. Parental satisfaction (4.9/6.0) and child satisfaction (3.8/5.0) were high (see Table 1).

**Table 1:** Parent (n=10) and child (n=10) satisfaction survey results.

Survey items		Rating score						
		Mean (SD)	1	2	3	4	5	6
**Parent items 1 (responses ranged from 1=Strongly Disagree to 6=Strongly Agree)**								
	Seeing my child’s weight on a graph every week helped me make better food choices for him/her.	4.4 (1.2)	0	0	3	3	1	3
	My child was willing to step on the bathroom scale once per week.	5.6 (0.8)	0	0	0	2	0	8
	My child was willing to wear a pedometer every day.	4.0 (2.4)	4	0	0	0	0	6
	Tracking my child’s steps each day helped him/her reach physical activity goals.	4.5 (1.7)	0	2	2	0	1	5
	Tracking the foods my child ate helped him/her reach weight goals.	5.1 (1.2)	0	0	2	1	1	6
	The healthy tips my child and I received helped me make healthy lifestyle changes for my child.	5.4 (0.9)	0	0	1	0	3	6
	The information I received in my health tips helped me make healthy lifestyle changes for my family & myself.	5.3 (0.8)	0	0	0	2	3	5
	I enjoyed the individual time talking with my counselor.	5.8 (0.4)	0	0	0	0	2	7
	The amount of time talking with my interventionist was enough.	5.7 (0.5)	0	0	0	0	3	7
	I would have liked to spend more time talking with my interventionist.	1.8 (0.9)	5	2	3	0	0	0
	I enjoyed meeting with my counselor remotely (by phone call or video chat on my smartphone).	5.8 (0.4)	0	0	0	0	2	8
**Parent items 2 (responses ranged from 1=Not helpful to 6=Very Helpful)**								
	Learning about the importance of self-monitoring how much we eat and our activity.	5.7 (0.5)	0	0	0	0	3	7
	Learning about portion control.	5.7 (0.5)	0	0	0	0	3	7
	Learning about choosing the right foods for you and your child.	5.4 (0.7)	0	0	0	1	4	5
	Learning about how to build good social support.	5.4 (0.9)	0	0	1	0	3	6
	Learning about fat, protein, and carbohydrates.	5.5 (0.5)	0	0	0	0	5	5
	Learning about how to overcome barriers to being healthy.	5.4 (0.9)	0	0	1	0	3	6
	Learning about how to make better choices when eating outside the home.	5.7 (0.5)	0	0	0	0	3	7
	Learning how to make healthy choices on special occasions such as birthday parties and school functions.	5.3 (0.6)	0	0	0	1	5	4
	Learning about healthy eating plans for the whole family, like the Stoplight approach to healthy eating.	5.7 (0.5)	0	0	0	0	3	7
	Learning about how much physical activity is recommended for me and my child.	5.5 (0.5)	0	0	0	0	5	5
	Taking a closer look at why we eat.	5.3 (0.6)	0	0	0	1	5	4
	Learning about healthy beverage choices for me and my child.	5.2 (1.5)	1	0	0	0	3	6
**Child items (responses were 1=No; 2=I don’t think so; 3=Maybe; 4=I think so; 5=Yes)**								
	I liked wearing my pedometer.	3.1 (0.4)	2	0	5	1	2	N/A
	I liked seeing how many steps I can get each day.	4.3 (0.3)	0	1	1	2	8	N/A
	The pedometer was easy to use.	3.5 (0.5)	1	2	1	3	3	N/A
	I tried to move more.	3.5 (0.5)	2	0	2	3	3	N/A
	I liked talking to [interventionist] about eating healthy foods and being more active.	4.0 (0.4)	1	0	2	2	5	N/A
	I tried to eat healthier foods.	4.1 (0.4)	1	0	2	1	6	N/A
	I tried new healthy foods that I had not tried before.	3.8 (0.5)	1	1	2	1	5	N/A
	I ate less candy.	3.3 (0.5)	2	2	1	1	4	N/A
	I drank less soda.	3.7 (0.5)	1	1	2	2	4	N/A
	I talked with my parents about eating healthier foods.	2.7 (0.5)	3	1	3	2	1	N/A
	Getting on the scale once a week was easy.	4.2 (0.4)	1	0	1	2	6	N/A

### Study 2

Nearly all respondents indicated that SafeCare families would benefit from learning how to eat more healthily and be more active (71/74, 96% and 68/74, 92%, respectively), and many (57/72, 79%) perceived that families would benefit from a program for child weight management. Most providers indicated that they were interested in learning how to deliver nutrition and physical activity information to their families (70/74, 95% and 60/74, 81%, respectively). About 80% (59/74) of providers reported that they and their SafeCare families would benefit from digital tools to support child weight management (see Table 2).

**Table 2:** Mean feasibility ratings reported by SafeCare providers (N=74), followed by the number (n) of providers who endorsed each rating from Strongly Disagree (1) to Strongly Agree (4).

Survey items	Mean (SD)	Strongly Disagree, n	Disagree, n	Agree, n	Strongly Agree, n
The parents I work with have regular access to healthy foods.	2.5 (0.7)	7	25	39	3
The parents I work with and their families would benefit from learning more about how to eat healthy.	3.3 (0.6)	1	2	44	27
I would be interested in learning how to deliver nutrition information to the parents I work with.	3.4 (0.6)	0	4	38	32
Most of the parents I work with or their families would benefit from weight loss or better weight management.	2.9 (0.8)	1	25	28	20
I would be interested in learning how to deliver weight management information to the parents I work with.	2.9 (0.9)	5	20	25	24
The parents I work with and their families would benefit from learning more about healthy levels of physical activity and exercise.	3.3 (0.6)	0	6	43	25
I would be interested in learning how to deliver information on physical activity to the parents I work with.	3.1 (0.7)	0	14	38	22
The parents I work with would benefit from a home visiting program designed to improve the body weight and health of young children in the home.^a^	3.0 (0.7)	1	14	40	17
The parents I work with would benefit from mobile health tools (smartphones, online dashboards) designed to improve their diet, activity levels, body weight, and health.	3.1 (0.7)	0	14	42	18
I would be interested in receiving support via mobile health tools (smartphones, online dashboards) to help me deliver health and weight management information to the parents I work with and their families.	3.1 (0.7)	0	15	34	25

^a^N=72 due to missing responses.

## Discussion

### Principal Findings

In this one-arm small pilot study, an mHealth weight management program significantly reduced children’s BMIz, and both parents and children had high levels of satisfaction. These data complement and build upon the prior DRIVE in-person home-based weight management program by integrating digital tools including telehealth counseling sessions, a wireless scale that enabled a child-tailored weight graph, and a pedometer to track child physical activity. Further, the survey of SafeCare providers indicated that providers perceive a need for this type of family-based weight management program and expect that their families will find remotely delivered content and digital tools to be acceptable.

Collectively, these preliminary data suggest that a weight management program delivered to parent/child dyads may be successful when implemented alongside a parenting program, such as SafeCare, via an mHealth platform. These data contribute to the burgeoning evidence that telehealth may be useful as adjunctive to in-person pediatric weight management. A nonrandomized comparative effectiveness study of 100 adolescents participating in a 2-year weight management program compared in-person plus telehealth versus in-person only and observed similar BMI outcomes, attendance rates, and acceptability among families and healthcare providers across the two groups [21]. Digital tools may not only remove barriers to transportation and scheduling for in-person care delivery but also expand reach of interventions to areas that are less likely to have access to multi-disciplinary care, particularly to families who are low income with limited resources such as those served by SafeCare agencies.

A key benefit for the remote delivery of weight management counseling is to increase accessibility to families, especially in more rural areas. However, the family must have the necessary equipment including an internet-enabled device (eg, smartphone, tablet, or computer that is connected to the internet via either a cellular network or WiFi). A recent study of the virtual delivery of SafeCare indicated that many families experienced limited broadband access and technology fatigue, resulting in the need to deliver shorter counseling sessions less than 30 minutes in length [8]. Online interactions may also lessen rapport between the provider and family due to limited ability to see nonverbal cues such as body language. A prior study of a hybrid version of SafeCare, including both face-to-face and virtual sessions, indicated that technology assistance offered efficiencies to the providers in terms of preparation for sessions, but the provider spent more time engaged in rapport-building activities with the family when delivered remotely [22].

Importantly, the parents and children in the pilot study expressed high levels of satisfaction with the remotely delivered program. Children rated satisfaction with talking to their counselor about eating healthier foods as higher than talking with their parent about eating healthier foods, highlighting the effectiveness of remote counseling but the need for further support of the parent-child interaction regarding healthy behavior change. Our findings expand upon a prior study of 360 children and parents randomized to a telehealth family-centered weight management arm in which parents had high levels of engagement and satisfaction with a combination of interactive text messaging and telehealth video calls [23]. A systematic review indicated noninferiority in children’s weight status improvement in telehealth versus in-person treatment delivery, with no difference in attrition rates and consistently high parental satisfaction with telemedicine [24].

Further, these findings add information that SafeCare providers report they are willing and interested in being trained in delivering weight management and believe their families would find this approach with digital tools acceptable. The integration of weight management into a previously existing structured parenting program provides an opportunity for large-scale and rapid dissemination. Families who receive services from SafeCare are often experiencing cumulative risk and have many needs, some of which are not directly related to abuse or neglect. Because SafeCare is broadly disseminated, training providers who already have a connection with these vulnerable families can be a vehicle for delivery of prevention programming that targets other public health issues a family may be experiencing. Should DRIVE prove beneficial, it could be offered as a module of additional services that families could receive. As detailed by the survey of SafeCare providers, there is a perceived need for services such as DRIVE, and SafeCare providers are willing to be trained to provide these services.

The pilot study observed a –0.14 reduction in BMIz (–0.18 among youth who were overweight or had obesity) with 8 counseling sessions delivered over a 13-week period. This reduction is greater than a prior 12-month study that observed –0.09 BMIz among children receiving both enhanced standard of care arm and individualized telehealth coaching (text messages 2x/week and telephone/video sessions every other month) [25] and greater than similar family-based weight management interventions according to a recent Cochrane review of interventions that lasted 6 months or longer [26]. However, the total contact hours did not meet the US Preventive Services Task Force recommendations of at least 26 hours to align with prior efficacious interventions [2], and the BMIz reduction did not meet previously suggested threshold of –0.25 for cardiometabolic improvement [27]. Importantly, in the pilot study, only 4 of the 10 children had obesity and an additional 4 were overweight, and it is not known if these children had cardiometabolic dysregulation. Future work should follow children over a longer time course to determine if BMIz reductions are sustained and accrue longer-term health benefits.

Increasing the dosage of telehealth weight counseling may increase weight loss. For example, a prior study of Kurbo, a commercially available weight management program delivered over a mobile app with video coaching sessions, showed that children who engaged in more telehealth coaching sessions over a longer duration had greater weight loss compared to those with less engagement [28], albeit the level of engagement was self-selected by the family and not randomly assigned. Similarly, a three-arm nonrandomized cohort study observed significantly reduced BMIz among children who opted into a multicomponent technology intervention that included family-based behavioral group treatment, a digital tablet with a fitness tracking app, and individually tailored telehealth coaching sessions, compared to those who received only the group counseling or the group counseling with fitness app [29]. Programs must strike a balance between families’ compliance/adherence to counseling sessions and expected weight reduction. The convenience of telehealth and digital tools may enable a sufficient amount of engagement that is both effective and acceptable to families.

### Limitations

A limitation of these studies is the one-arm design of the pilot feasibility study without a control or comparator condition and the need for further verification in a larger randomized controlled trial. It is possible that BMIz fluctuations were influenced by maturation bias or regression to the mean [30], though the observed effect size was similar to prior pediatric weight management interventions [26]. Another limitation is the use of BMIz to examine change over time, as researchers have identified concerns with z-score for children with a BMI above the 97th percentile [31]. However, only one child in the sample had a BMI exceeding 97th percentile, so it was determined that this metric was appropriate.

### Implications for Research and Practice

Our preliminary work demonstrates that DRIVE is an efficacious childhood weight management program capable of being delivered as a module within existing home-based programs, such as SafeCare, and adaptation of DRIVE to include mHealth would benefit both families and SafeCare providers. Families adhered to and were highly satisfied with the telehealth counseling sessions, the wireless scale and weight graph to track child weight, and the pedometer to track child physical activity. These findings are consistent with emerging research documenting that families are responding well to SafeCare delivery via technology, a delivery approach that was implemented as a result of the COVID-19 pandemic [8]. Integrating the intervention into a comprehensive smartphone app or website may enable a more seamless delivery system of both self-monitoring tools and ongoing remote interaction with the counselor. There are many future areas of investigation for mHealth DRIVE, including measuring the effects on weight-related behaviors including dietary intake and physical activity, examining specific feature utilization of the intervention components (such as sharing photos of food preparation with the counselor) and how this relates to the effectiveness of the intervention, and the extent to which the relationship with the counselor drives health outcomes in the family.

Digital tools may present an opportunity for a hybrid approach to blend in-person care with remotely delivered care, bridging the gap between counseling sessions by equipping parents and children with tools to continue their self-monitoring and assist them in implementing the health lessons into their daily lives. SafeCare providers overwhelmingly indicate the perceived need, and willingness to deliver, such a program. Our future work aims to test the feasibility and effectiveness of the mHealth DRIVE program over a longer term to manage children’s weight and improve health-related parenting skills within the context of SafeCare’s telehome visit delivery model. The ultimate goal is to package a turn-key weight management program for families of children with obesity, deployed using mHealth tools for wide-scale dissemination.

## Abbreviations

DRIVE: Developing Relationships that Include Values of Eating and Exercise
